# Silibinin alleviates intestinal inflammation via inhibiting JNK signaling in *Drosophila*

**DOI:** 10.3389/fphar.2023.1246960

**Published:** 2023-09-14

**Authors:** La Yan, Juanyu Zhou, Lu Yuan, Jinbao Ye, Xudong Zhao, Gang Ren, Haiyang Chen

**Affiliations:** ^1^ Laboratory of Metabolism and Aging Research, Frontiers Science Center for Disease-Related Molecular Network, State Key Laboratory of Respiratory Health and Multimorbidity and National Clinical Research Center for Geriatrics, West China Hospital, Sichuan University, Chengdu, Sichuan, China; ^2^ Department of Targeting Therapy and Immunology and Laboratory of Animal Tumor Models, Cancer Center and National Clinical Research Center for Geriatrics and Frontiers Science Center for Disease-related Molecular Network, West China Hospital, Sichuan University, Chengdu, Sichuan, China; ^3^ Research Center of Natural Resources of Chinese Medicinal Materials and Ethnic Medicine, Jiangxi University of Chinese Medicine, Nanchang, Jiangxi, China

**Keywords:** silibinin, intestinal inflammation, intestinal stem cells, JNK, *Drosophila melanogaster*

## Abstract

Inflammatory bowel diseases (IBDs) are characterized by chronic relapsing intestinal inflammation that causes digestive system dysfunction. For years, researchers have been working to find more effective and safer therapeutic strategies to treat these diseases. Silibinin (SIL), a flavonoid compound extracted from the seeds of milk thistle plants, possesses multiple biological activities and is traditionally applied to treat liver diseases. SIL is also widely used in the treatment of a variety of inflammatory diseases attributed to its excellent antioxidant and anti-inflammatory effects. However, the efficacy of SIL against IBDs and its mechanisms remain unclear. In this study, using *Drosophila melanogaster* as a model organism, we found that SIL can effectively relieve intestinal inflammation caused by dextran sulfate sodium (DSS). Our results suggested that SIL supplementation can inhibit the overproliferation of intestinal stem cells (ISCs) induced by DSS, protect intestinal barrier function, acid-base balance, and intestinal excretion function, reduce intestinal reactive oxygen species (ROS) levels and inflammatory stress, and extend the lifespan of *Drosophila*. Furthermore, our study demonstrated that SIL ameliorates intestinal inflammation via modulating the c-Jun N-terminal kinase (JNK) signaling pathway in *Drosophila*. Our research aims to provide new insight into the treatment of IBDs.

## 1 Introduction

Inflammatory bowel diseases (IBDs), which mainly include Crohn’s disease and ulcerative colitis, are chronic, relapsing-remitting, immune-mediated intestinal disorders with a complex etiology and multiple risk factors such as genetics, environment, and immunity (Torres et al., 2017; Ungaro et al., 2017). At present, the main therapies for IBDs are immunomodulatory agents and biological therapy, which aim to achieve symptom remission and long-term maintenance (Jeong et al., 2019; Chapman et al., 2020). However, the application of these agents is severely limited by low efficacy, side effects, and drug resistance (Neurath, 2017). Therefore, it is urgent to find safe and effective treatment strategies for IBDs.

Silibinin (SIL) is the major active component of silymarin, which is a flavonoid extract from milk thistle seeds (Biedermann et al., 2014). SIL possesses various biological activities, including antioxidant, anti-inflammatory, anticancer, and anti-fibrotic activities (Abenavoli et al., 2010; Bosch-Barrera and Menendez, 2015; Mizuno et al., 2020; Fallah et al., 2021). SIL has been reported to be an effective compound against multiple cancers, diabetes, Alzheimer’s disease, and hepatic diseases (Bosch-Barrera et al., 2017; Chu et al., 2018; Liu et al., 2019; Shen et al., 2019). Due to its excellent antioxidant and anti-inflammatory activities, SIL has been widely used in the treatment of inflammation-related diseases, such as skin inflammation, sepsis, and hepatitis (Kim et al., 2013; Liu et al., 2015; Federico et al., 2017; Sun et al., 2022). Accumulating evidence suggests that patients with IBDs have an increased risk of developing colorectal cancer, which is attributed to chronic intestinal inflammation (Beaugerie and Itzkowitz, 2015; Rajamaki et al., 2021). A previous study has shown that SIL can repress inflammation-associated colon cancer in mice (Li et al., 2019). However, the effect of SIL on intestinal inflammation treatment and its mechanism remain unclear.

IBDs are characterized by relapsing mucosal inflammation causing severe intestinal epithelial injury and subsequent intestinal barrier dysfunction (Atreya and Neurath, 2015). The intestinal epithelial barrier depends on intestinal stem cells (ISCs), a group of pluripotent cells capable of self-renewal and differentiation, for regeneration and homeostasis maintenance (Barker, 2014). Under homeostatic conditions, ISCs self-renew every few days to maintain intestinal epithelial function (Peterson and Artis, 2014). When exposed to harmful stimuli, ISCs are rapidly activated to proliferate and differentiate to repair the damaged intestinal epithelium (Bankaitis et al., 2018). Several signaling pathways, such as Wnt signaling (Clevers et al., 2014), Janus-activated kinase/signal transducer and activator of transcription (JAK/STAT) signaling (Herrera and Bach, 2019), and MAPK pathway (Kabiri et al., 2018), are involved in regulating ISC proliferation and intestinal homeostasis. In recent years, studies have shown that promoting intestinal mucosal healing is beneficial to the treatment of IBDs, and ISC-driven rebuilding of the mucosa is effective for IBDs therapy (Shah et al., 2016; Villablanca et al., 2022). Therefore, it is possible to treat IBDs by regulating the function of ISCs in the future.

*Drosophila melanogaster* is a well-established model organism for genetic research due to its high conservation, simple operation, and wide availability (Fox et al., 2020; Dow et al., 2022). In the last decades, *Drosophila* is increasingly recognized as a valuable model for the study of intestinal inflammation (Madi et al., 2021; Nagai and Yano, 2021). The *Drosophila* intestine is a multifunctional organ mainly composed of four types of cells: intestinal stem cells (ISCs), enteroblasts (EBs), absorptive enterocytes (ECs), and secretory enteroendocrine cells (EEs) (Miguel-Aliaga et al., 2018). ISCs can divide asymmetrically, with the basal daughter cells remaining ISCs to maintain the stem cell pool, while the apical daughter cells are considered EBs, which are further differentiated into ECs or EEs (Micchelli and Perrimon, 2006; Kabiri et al., 2018). Similar to the mammalian intestine, ISC proliferation in *Drosophila* is regulated by multiple signaling pathways, such as Wnt, c-Jun N-terminal kinase (JNK), epidermal growth factor receptor (EGFR), and JAK-STAT signaling pathways (Jiang et al., 2016; Herrera and Bach, 2021; Zhang C. et al., 2022). When *Drosophila* was exposed to harmful chemicals such as sodium dodecyl sulfate (SDS), dextran sulfate sodium (DSS), and bleomycin (BLM), the intestinal barrier integrity was impaired, leading to the development of intestinal inflammation along with an increased proliferation of ISCs and a shortened lifespan (Du et al., 2020; Zhang G. et al., 2022; Yang et al., 2022). Therefore, *Drosophila* was selected as a model organism to investigate the effect and mechanism of SIL in the treatment of intestinal inflammation.

In this study, we found that SIL alleviates *Drosophila* intestinal inflammation induced by DSS or BLM. Application of SIL can inhibit ISCs hyperproliferation, protect intestinal function, and extend the lifespan of *Drosophila* with intestinal inflammation. In the mechanisms, our study demonstrated that SIL alleviates intestinal inflammation through the inhibition of the JNK signaling pathways.

## 2 Materials and methods

### 2.1 *Drosophila* strains and culture

The following *Drosophila* strains were used in this study: *esg-GFP/CyO* and *UAS-lacZ* lines were kindly provided by Dr. Allan Spradling; *esg* ^*ts*^ *-Gal4* line was generously gifted from Dr. Benjamin Ohlstein; *w* ^*1118*^ (BDSC #3605), *Canton-S* (BDSC #64349), *UAS-bsk* ^*DN*^ (BDSC #6409) and *UAS-EGFR* ^*DN*^ (BDSC #5364) were obtained from the Bloomington *Drosophila* Stock Center. All flies used in this study were mated females unless otherwise mentioned.

Flies were maintained on standard cornmeal-agar medium (the recipe is: 80 g sucrose, 50 g cornmeal, 20 g glucose, 18.75 g yeast, 5 g agar, 30 mL propionic acid, and 1 L water) at 25°C and 60% humidity under a 12/12 h light/dark cycle. Gene overexpression driven by the *esg* ^*ts*^ *-Gal4 Drosophila* line was repressed at 18°C and activated at 29°C.

### 2.2 Drugs treatment

#### 2.2.1 SIL treatment

SIL (Macklin, Shanghai, China, #S817883) was first dissolved in DMSO (Dimethyl Sulfoxide, BioFroxx, #1084ML100), then added to standard food to prepare different concentrations. Freshly hatched flies were collected and cultured in the medium with different concentrations of SIL.

#### 2.2.2 DSS and BLM treatment

Chromatography paper was cut to a size of 3.7 cm × 5.8 cm and placed in tubes, the paper was completely moistened with 500 µL of 5% sucrose or 7% DSS (Yeasen Biotechnology, Shanghai, China, #60316ES80, dissolved in 5% sucrose). 7-day-old flies were collected, starved for 1 h in empty tubes, and then cultured in tubes containing 5% sucrose or 7% DSS for 3 days before dissection. BLM (Aladdin, Shanghai, China, #B107423) was applied at a concentration of 25 µg/mL (dissolved in 5% sucrose) and flies were cultured for 24 h.

### 2.3 Immunofluorescence

*Drosophila* intestines were dissected in cold PBS (Phosphate Buffered Saline, Servicebio, #G0002), fixed in 4% paraformaldehyde for 30 min at room temperature, and then rinsed three times with 0.1% PBST (Triton X-100, BioFroxx, #1139ML100) for 10 min each. Tissues were immersed in the primary antibody solution and incubated overnight at 4°C. The next day, the intestines were rinsed three times with 0.1% PBST for 10 min each. Next, the tissues were incubated with the secondary antibody solution for 2 h at room temperature and then rinsed three times with 0.1% PBST for 10 min each. Finally, the intestines were soaked with the anti-fluorescence quenching agent and placed on slides for sealing and storage. Immunofluorescence images were taken with a Leica TCS-SP8 confocal microscope and processed with Application Suite X, Adobe Illustrator, and ImageJ software. Information on the antibodies used in this study is shown in Supplementary Table S1.

### 2.4 Smurf assay

The smurf assay was performed to test the integrity of the *Drosophila* intestinal barrier (Rera et al., 2011). The blue food dye (Spectrum Chemical Manufacturing Corp, Shanghai, China, #FD110) was added to the standard medium to a concentration of 2.5% (wt/vol). *Drosophila* treated with different drugs (fed with or without SIL) were starved for 1 h and then cultured in the medium for 12 h. When the *Drosophila* intestinal barrier is intact, the blue food dye is confined to the digestive tract. We refer to flies that leak blue dye into tissues outside the intestine as smurf (+) *Drosophila*.

### 2.5 Bromophenol blue assay

The bromophenol blue assay was performed to determine whether the acidic state of the copper cell region (CCR) in the *Drosophila* intestine is normal (Li et al., 2016). Briefly, 200 µL of 2% bromophenol blue solution (Sigma, #B5525, dissolved in 5% sucrose) was added to the surface of the standard medium and used after the solution was completely absorbed. The flies were starved for 1 h and cultured in food containing bromophenol blue solution for 24 h. The intestines were dissected and photographed immediately to prevent carbon dioxide from changing the color rendering results of bromophenol blue. When the CCR is acidic, the bromophenol blue appears yellow, which we called “Homeostasis”; if the CCR region is non-acidic, the bromophenol blue appears blue, which we called “Perturbed”.

### 2.6 *Drosophila* excretion assay

The *Drosophila* excretion assay was performed to measure the excretory function of the *Drosophila* intestine (Cognigni et al., 2011). Add 200 µL of 2% bromophenol blue solution to the surface of the medium and leave to allow the food to fully absorb. Cut the chromatography paper into a size of 3.7 cm × 5.8 cm and placed it on the side wall of the food tube. Be careful that the paper cannot touch the food with the bromophenol blue solution. The flies were starved for 1 h and cultured in the food for 24 h. The paper was taken out, and the droppings of flies were imaged with a Leica M205 FA stereomicroscope.

### 2.7 Lifespan assay

For the lifespan assay under DSS application, put a 3.7 cm × 5.8 cm chromatography paper in the tube and completely wet the paper with 7% DSS (dissolved in 5% sucrose). A total of 100 flies hatched within 48 h were collected and randomly placed into four DSS tubes with or without SIL. The dead flies were recorded every day and the paper was replaced. Repeat the experiment three times.

For the lifespan assay under BLM application, the BLM was dissolved in the standard medium to a concentration of 5 µg/mL. A total of 100 flies hatched within 48 h were randomly placed into four BLM food tubes with or without SIL. The dead flies were recorded once a day and the food was replaced. The experiment was repeated three times.

### 2.8 Dihydroethidium (DHE) staining

The DHE staining was performed to detect the level of ROS in the *Drosophila* intestine (Hochmuth et al., 2011). The intestines were dissected and incubated with DHE (MKbio, Shanghai, China, #MX4812) for 20 min at room temperature, then rinsed 3 times with PBS. Tissues were fixed with 4% paraformaldehyde at room temperature for 30 min and rinsed 3 times with PBS, and finally soaked with the anti-fluorescence quench agent. Images were taken immediately after DHE staining, and ImageJ software was used to calculate fluorescence intensity.

### 2.9 Real-time quantitative PCR

The *Drosophila* guts were dissected, frozen with liquid nitrogen, and ground immediately. Total RNA was extracted with an RNA-easy Isolation Reagent (Vazyme, Nanjing, China, #R701-01) and reverse transcribed into cDNA with an *Evo M-MLV* RT Kit (Accurate Biology, #AG11711). RT-qPCR was performed with a CFX96TM Real-time PCR System (BIO-RAD, America) using ChamQ Universal SYBR qPCR Master Mix (Vazyme, Nanjing, China, #Q711-02). The *Rp49* gene was used as an internal control, and the relative mRNA expression of genes was calculated using the 2^−ΔΔCT^ method. The primer sequences used in this study are listed in Supplementary Table S2.

### 2.10 Network pharmacology

The corresponding targets of SIL were obtained from the PharmMapper database and screened the targets with Norm Fit >0.5 (Wei et al., 2023). The targets of IBD were searched by the OMIM, TTD, DrugBank, GeneCards, and DisGeNET databases using “inflammatory bowel disease” as the keyword (Zu et al., 2021). The gene names of these targets were obtained from the Uniprot database after removing the duplicates (Pei et al., 2023).

The common targets of SIL and IBD were analyzed by the Venny 2.1.0 database to predict the potential targets of SIL against IBD (He et al., 2023). These common targets were imported into the STRING database, the minimum required interaction score was set to 0.7 and the isolated targets were removed to obtain the protein-protein interaction (PPI) network (Wu et al., 2023).

Kyoto Encyclopedia of Genes and Genomes (KEGG) enrichment analysis of the common targets was performed using the DAVID database (He et al., 2023). The enrichment results of KEGG pathways were visualized by the Bioinformatics platform (He et al., 2023).

### 2.11 Molecular docking

The 3D structure of SIL in the SDF file was obtained from the PubChem database, converted to a mol2 file via Open Babel 3.1.1 software, used Autodock MGL Tools 1.5.7 to add hydrogen bonds, detect the root, and set rotatable bonds, then saved as PDBQT format (Li et al., 2023). The 3D structures of the key target proteins obtained from the PDB database were exported to the PDB file. The water molecules and excess inactive ligands were removed by PyMOL software. The proteins were hydrogenated and charged into AutoDockTools 1.5.7 software and exported to PDBQT format (Li et al., 2023). The molecular docking was performed using AutoDock Vina 1.1.2 software and the results were visualized by Discovery Studio 2016 Client software (Ersoy et al., 2023).

### 2.12 Statistical analyses

All experiments were performed with at least three replicates. All data are presented as means ± standard deviations (SD). The statistical significance was analyzed using GraphPad Prism version 8.0. The two-tailed Student’s t-test was performed to analyze differences between groups. The lifespan assays were tested for significance with a log-rank test. *P* < 0.05 was considered statistically significant, *p < 0.05, **p < 0.01, ***p < 0.001 and ****p < 0.0001. Non-significance (ns) represents *p* > 0.05.

## 3 Results

### 3.1 SIL prevents ISC overproliferation in *Drosophila* with intestinal inflammation

**FIGURE 1:**
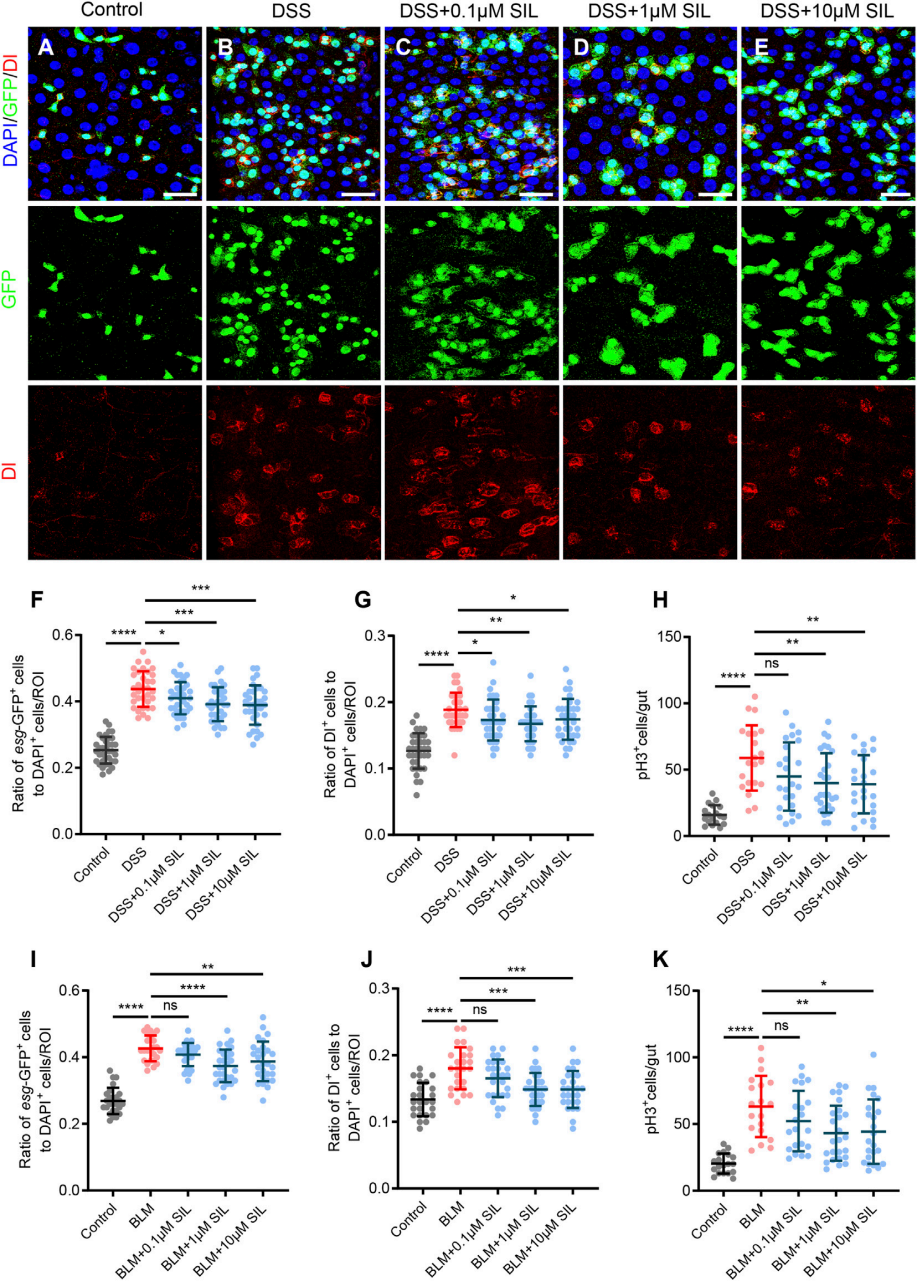
SIL inhibits DSS or BLM-induced ISC overproliferation. **(A–E)** Immunofluorescence images of *Drosophila* (*esg-GFP/CyO*) posterior midguts of GFP and Dl staining in control flies and 7% DSS stimulated flies supplemented with or without SIL. Three concentrations of SIL were studied: 0.1, 1, and 10 µM. GFP: green, ISCs and progenitor cells marker; Dl: red, ISCs marker; DAPI: blue, nuclei. Scale bars represent 25 µm. **(F,G)** Quantification of the ratio of *esg*-GFP positive cells and Dl positive cells to DAPI positive cells per ROI (region of interest) in DSS experiments (*n* = 36, 34, 35, 33, 34 from left to right). **(H)** Quantification of the number of pH3 positive cells in the whole guts of flies in DSS experiments (*n* = 18, 21, 24, 27, 24). pH3: phosphorylated-histone 3, a specific marker for proliferating cells. **(I,J)** Quantification of the ratio of *esg*-GFP positive cells and Dl positive cells to DAPI positive cells per ROI in BLM experiments (*n* = 25, 28, 30, 28). **(K)** Quantification of the number of pH3 positive cells in the whole guts of flies in BLM experiments (*n* = 19, 21, 25, 23). Data are represented as means ± SD. Student’s *t*-tests, ns represents *p* > 0.05, *p < 0.05, **p < 0.01, ***p < 0.001, and ****p < 0.0001.

When the *Drosophila* intestine was exposed to noxious stimuli, the barrier integrity was impaired, leading to intestinal inflammation along with an increased proliferation of ISCs (He et al., 2022; Yang et al., 2022). To investigate the effect of SIL on the proliferation of ISCs in *Drosophila* caused by DSS insults, we used a reporter line, *esg-GFP/CyO*, whose ISCs and progenitor cells could be detected by expressing green fluorescent protein (GFP). In addition, we observed the ISCs by Delta (Dl) staining and the dividing ISCs by phosphorylated-histone 3 (pH3) staining (Zhou et al., 2023). The results showed that when stimulated by DSS, the proliferation of ISCs was significantly enhanced, which was manifested by the increase in the number of *esg*-GFP^+^, Dl^+^, and pH3^+^ cells (Figures 1A, B,F–H). We found that SIL supplementation significantly reduced the DSS-induced increase in the number of *esg*-GFP^+^, Dl^+^, and pH3^+^ cells. Furthermore, of the three concentrations tested (0.1, 1, and 10 µM), 1 µM SIL was the most effective, showing the least *esg*-GFP^+^, Dl^+^, and pH3^+^ cells (Figures 1C–H). To enhance the persuasiveness and comprehensiveness of these findings, we also applied Bleomycin (BLM), another reagent that induces intestinal damage by causing DNA damage (Amcheslavsky et al., 2009), and obtained the same results (Supplementary Figures S1A–E, Figures 1I–K). Based on these results, we selected 1 µM SIL as the optimal concentration for conducting further experiments. A previous study reported that the intestinal damage caused by DSS or BLM would return to normal within 3 days (rec-3d) (Du et al., 2020). Our results suggest that SIL supplementation inhibits DSS-induced overproliferation of ISCs without affecting eventual intestinal repair (Supplementary Figures S1F, G). All these results suggested that SIL can inhibit the overproliferation of ISCs during intestinal inflammation in *Drosophila*.

### 3.2 SIL protects *Drosophila* intestinal function from inflammatory damage

**FIGURE 2:**
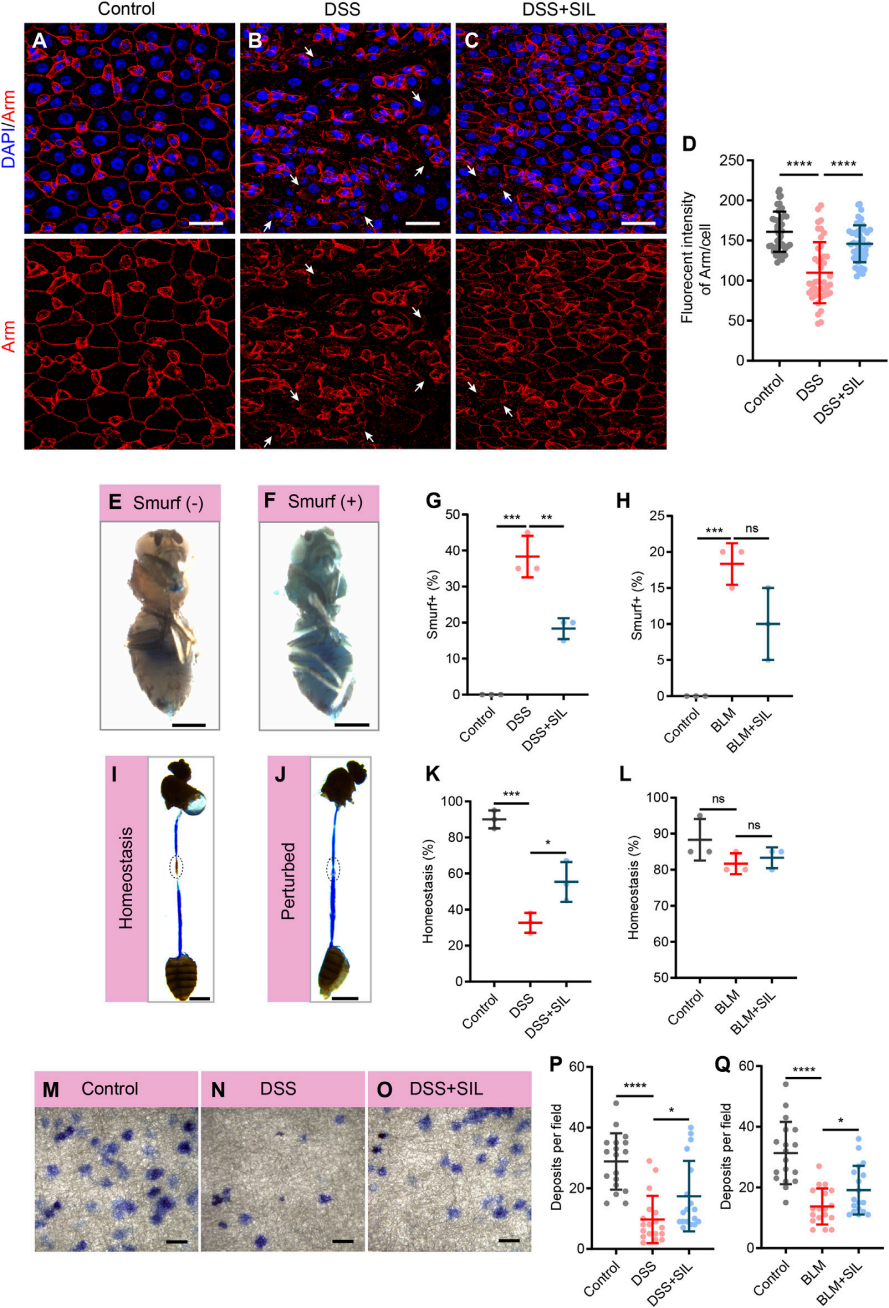
SIL prevents stimuli-induced intestinal dysfunction. **(A–C)** Immunofluorescence images of *Drosophila* (*esg-GFP/CyO*) posterior midguts of Armadillo (Arm) staining in control flies and 7% DSS stimulated flies supplemented with or without 1 µM SIL. Arm: red, cell membrane maker. White arrows indicate irregular Arm staining. Scale bars represent 25 µm. **(D)** Quantitation of Arm fluorescence intensity per cell (*n* = 46, 42, 47). **(E,F)** Representative images of non-smurf and smurf fly. Scale bars represent 0.5 mm. **(G)** Quantification of the percentage of smurf (+) flies in DSS experiments. Three independent experiments were performed, each group included 20 flies. **(H)** Quantification of the percentage of smurf (+) flies in BLM experiments. **(I,J)** Representative images of acidic (Homeostasis) and non-acidic (Perturbed) CCR in flies. Circles indicate the CCR. Scale bars represent 1 mm. **(K)** Quantification of the percentage of Homeostasis flies in DSS experiments. Three independent experiments were performed, each group included 20 flies. **(L)** Quantification of the percentage of Homeostasis flies in BLM experiments. **(M–O)** Representative images of excretion deposits in control flies and 7% DSS stimulated flies supplemented with or without 1 µM SIL. Scale bars represent 2 mm. **(P)** Quantification of the number of deposits in DSS experiments (*n* = 18, 18, 18). **(Q)** Quantification of the number of deposits in BLM experiments (*n* = 18, 18, 18). Data are represented as means ± SD. Student’s *t*-tests, ns represents *p* > 0.05, *p < 0.05, **p < 0.01, ***p < 0.001, and ****p < 0.0001.

The intestine is an important organism for the digestion and absorption of food, the metabolism and elimination of waste products, and the immune defense (Funk et al., 2020). When the intestine suffers from inflammation, these physiological functions are disrupted. As our results suggested that SIL can repress the overproliferation of ISCs induced by DSS or BLM, and ISCs play an important role in the maintenance of intestinal functions, we wanted to further investigate the effect of SIL on intestinal functions under inflammation. We performed Armadillo (Arm) staining and smurf assay to determine the barrier integrity of the *Drosophila* intestinal epithelium. When the intestine was damaged by inflammation caused by DSS or BLM, its barrier function was significantly impaired, as evidenced by the irregular shape of Arm staining and the increased proportion of smurf (+) *Drosophila* (fly that leaks blue dye into tissues outside the intestine) (Figures 2A, B, D–H, Supplementary Figures S2A, B). We found that SIL supplementation can relieve the impairment of barrier function due to inflammatory damage (Figures 2C–H, Supplementary Figure S2C).

The cells in the copper cell region (CCR) of the *Drosophila* intestine secrete acid to make the region acidic, and if the acidic state is disrupted, it indicates abnormal intestinal function (Dubreuil, 2004). We performed the bromophenol blue assay to detect whether the acidic state of the CCR is normal, if the region is acidic, bromophenol blue appears yellow, otherwise blue. The results revealed that SIL supplementation could protect the acidic state of the CCR from the damage of DSS or BLM (Figures 2I–L). Besides, we found that when the intestine is subjected to inflammation, its excretory function is impaired, and SIL supplementation protects the excretory function of the gut (Figures 2M–Q, Supplementary Figures S2D–F). Taken together, these results suggested that SIL supplementation protects *Drosophila* intestinal function from inflammatory impairment.

### 3.3 SIL alleviates intestinal inflammation and prolongs the lifespan of *Drosophila*

**FIGURE 3:**
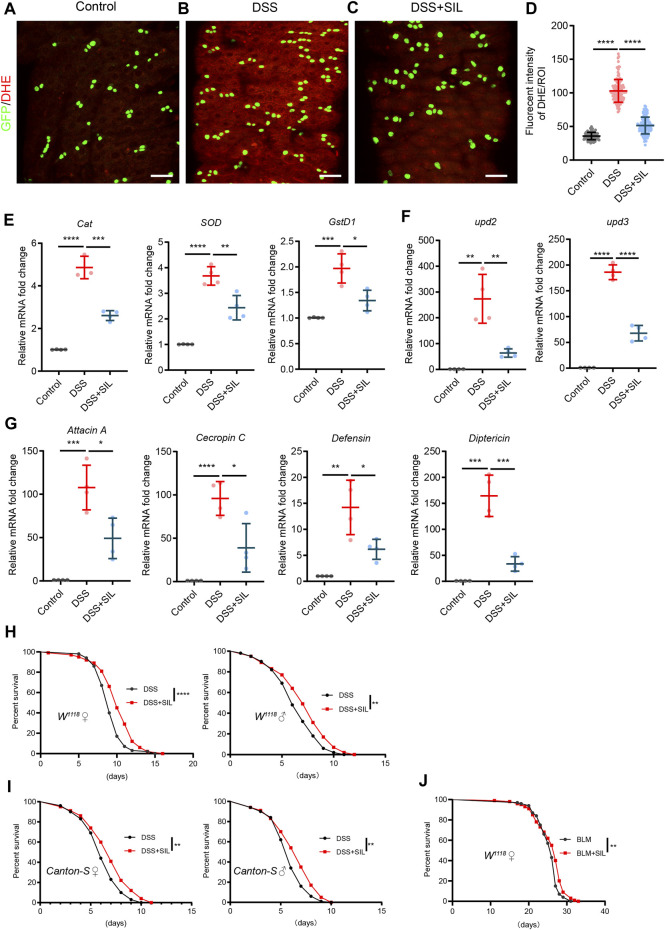
SIL alleviates oxidative stress, decreases the expression of AMPs, and extends the lifespan of *Drosophila.* **(A–C)** Immunofluorescence images of *Drosophila* (*esg-GFP/CyO*) posterior midguts of DHE staining in control flies and 7% DSS stimulated flies supplemented with or without 1 µM SIL. DHE: red, ROS maker. Scale bars represent 25 µm. **(D)** Quantitation of DHE fluorescence intensity per ROI (*n* = 88, 130, 119). **(E–G)** Quantification of relative mRNA expression of the antioxidant-related genes (*Cat, SOD,* and *GstD1*), AMPs (*Attacin A, Cecropin C, Defensin,* and *Diptericin*), and inflammatory cytokines (*upd2* and *upd3*) in the midguts of control flies and 7% DSS stimulated flies supplemented with or without 1 µM SIL. **(H,I)** Survival percentage of female (left) and male (right) *W* ^*1118*^ or *Canton-S* flies with (red curve) or without (black curve) 1 µM SIL supplementation under 7% DSS treatments. Three independent experiments were performed, each group included 100 flies. **(J)** Survival percentage of female *W* ^*1118*^ flies with (red curve) or without (black curve) 1 µM SIL supplementation under 5 µg/mL BLM treatments. Three independent experiments were performed, each group included 100 flies. Data are represented as means ± SD. Log-rank test for lifespan assay. Student’s *t*-tests, ns represents *p* > 0.05, *p < 0.05, **p < 0.01, ***p < 0.001, and ****p < 0.0001.

DSS causes inflammatory damage in the intestine, resulting in elevated levels of oxidative stress and activation of the immune response (Zhang G. et al., 2022; Dong et al., 2022). To investigate whether SIL can alleviate oxidative stress in DSS stimulated intestine, we used the fluorescent probe DHE to detect the ROS levels. We found that SIL supplementation significantly reduced the ROS induced by DSS (Figures 3A–D). In addition, RT-qPCR analyses revealed that SIL decreased the relative RNA expression level of antioxidant-related genes (*Cat*, *SOD*, and *GstD1*) in DSS-stimulated *Drosophila* midgut (Figure 3E). These results indicated that SIL supplementation decreased the high levels of oxidative stress in the *Drosophila* midgut induced by DSS.

Inflammatory damage to the intestinal epithelium activates the immune response and affects the expression of antimicrobial peptides (AMPs), a group of essential components of the intestinal epithelial barrier (Lazzaro et al., 2020). The results showed that when stimulated by DSS, the expression of AMPs (*Attacin A*, *Cecropin C*, *Defensin*, and *Diptericin*) and inflammatory cytokines [*upd2* and *upd3*, inflammatory IL-6-like cytokines in *Drosophila* (Jiang et al., 2009)] was significantly increased in the *Drosophila* midgut and SIL supplementation decreased the expression of AMPs, *upd2* and *upd3* (Figures 3F,G). The lifespan of *Drosophila* is shortened when exposed to chronic deleterious stimuli (Du et al., 2021; Zhang G. et al., 2022). We found that SIL supplementation dramatically prolonged the lifespan under DSS or BLM stimulation (Figures 3H–J). In conclusion, these results suggested that SIL can alleviate DSS-induced intestinal inflammation and extend the lifespan of *Drosophila*.

### 3.4 Network pharmacology analysis and molecular docking of SIL

**FIGURE 4:**
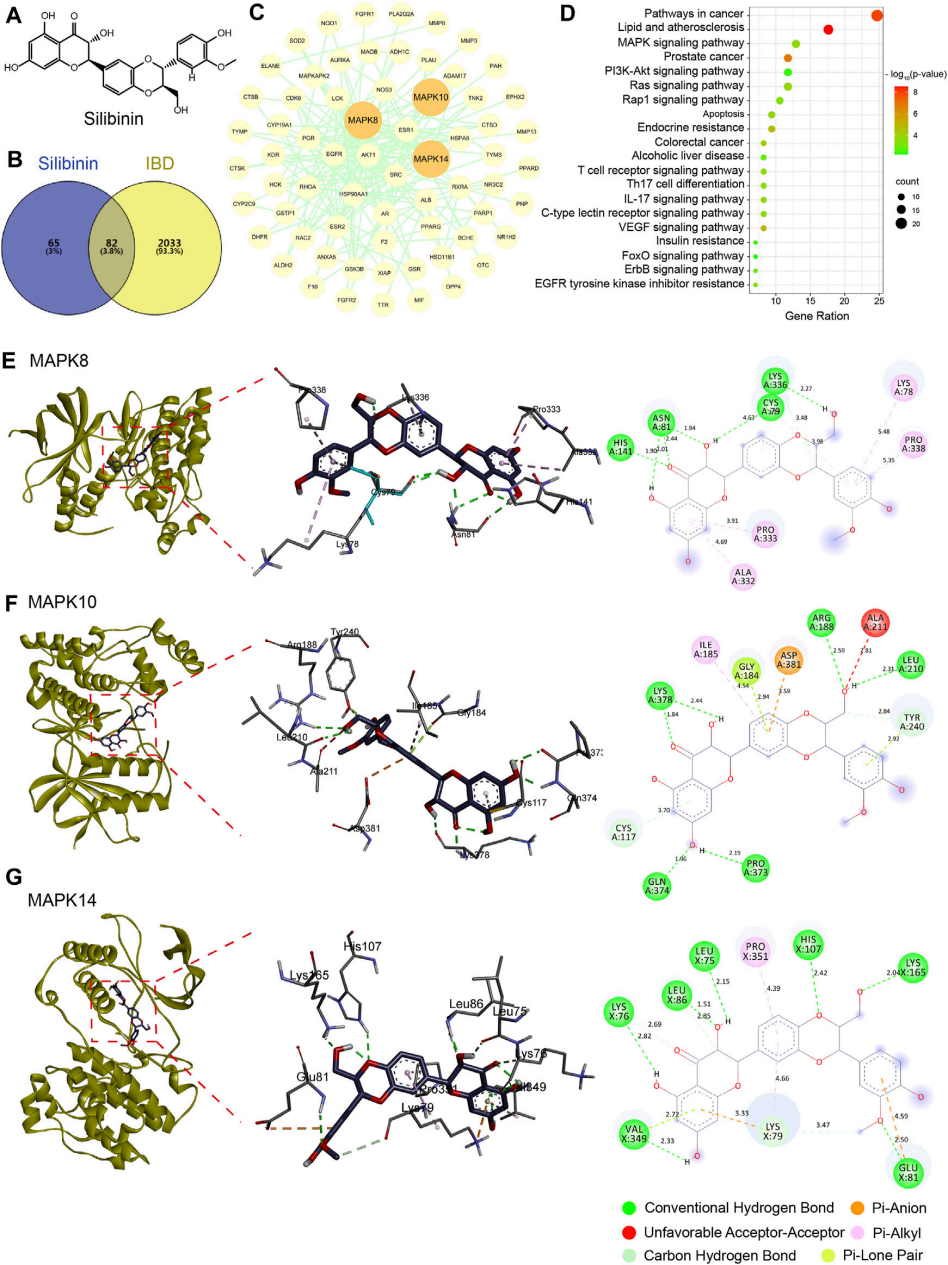
Network pharmacology and molecular docking. **(A)** The structured SDF file of SIL. **(B)** Venn diagram showing the intersection between SIL and IBD. **(C)** PPI network of SIL and IBD intersection targets. **(D)** Kyoto Encyclopedia of Genes and Genomes (KEGG) pathways bubble diagram. **(E–G)** Diagram of the molecular docking of SIL with three key targets, the binding energies for the targets docked into the SIL are MAPK8/JNK1 (−6.73 kcal/mol), MAPK10/JNK3 (−6.65 kcal/mol), MAPK14/p38-α (−5.87 kcal/mol).

Further, we investigated the mechanism by which SIL alleviates intestinal inflammation in *Drosophila* through network pharmacological analysis. 147 action targets for SIL were obtained from the PharmMapper and Uniprot databases and 2,115 targets for IBD were obtained from the OMIM, TTD, DrugBank, GeneCards, DisGeNET, and Uniprot databases (Figure 4B). Using the Venny database, we obtained 82 common targets for SIL and IBD (Figure 4B). These common targets were imported into the STRING database to obtain the protein-protein interaction (PIP) network (Figure 4C). We found that proteins (MAPK8, MAPK10, and MAPK14) involved in the JNK/p38 MAPK pathway, a classic stress signaling pathway (Hotamisligil and Davis, 2016), interact strongly with other proteins (Figure 4C). Besides, the Kyoto Encyclopedia of Genes and Genomes (KEGG) enrichment analysis was performed on the 82 common targets using the DAVID database to obtain the SIL-regulated signaling pathways, in which the genes related to the MAPK signaling pathway were significantly enriched (Figure 4D). To confirm the possible action targets and signaling pathways of SIL against IBD, molecular docking was performed to verify the accuracy of the network pharmacological predictions. The results showed that MAPK8 (JNK1), MAPK10 (JNK3), and MAPK14 (p38-α) possessed strong binding activity with SIL (Figure 4A, E–G). These results suggested that the JNK signaling pathway may be the mechanism by which SIL alleviates IBD.

### 3.5 SIL alleviates DSS-induced intestinal inflammation by repressing the JNK signaling pathway

**FIGURE 5:**
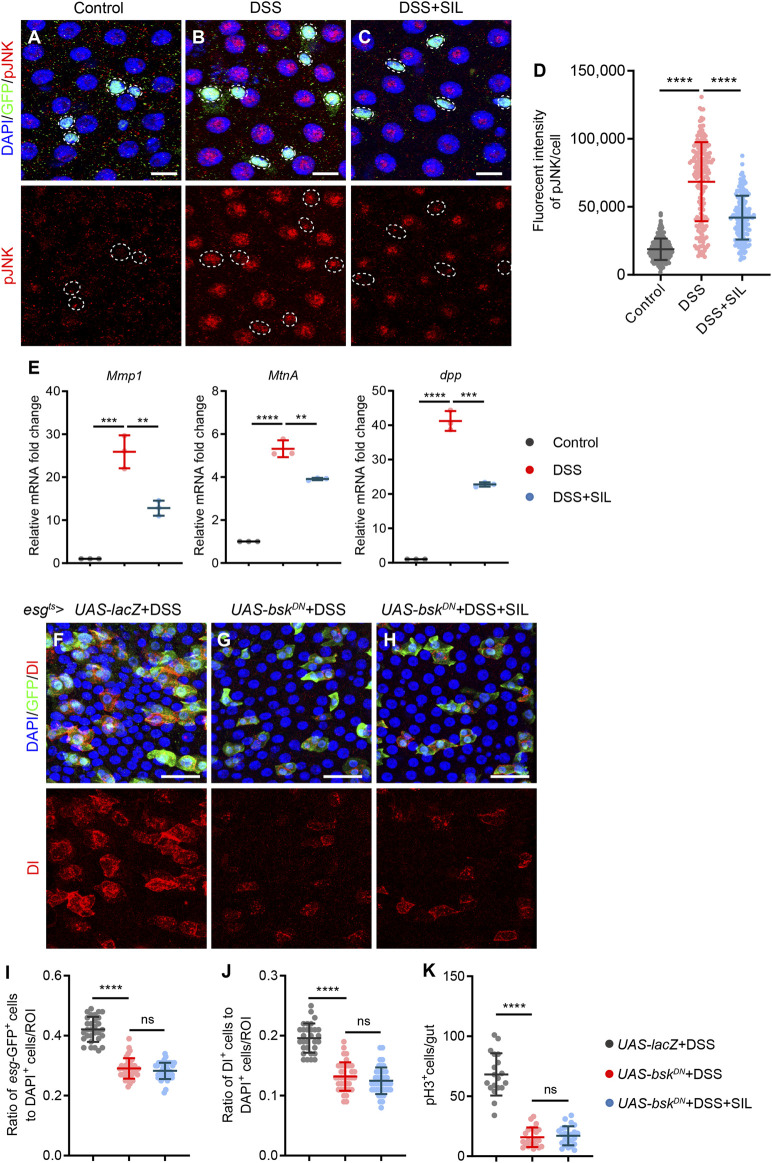
SIL inhibits the JNK signaling pathway. **(A–C)** Immunofluorescence images of *Drosophila* (*esg*-GFP/CyO) posterior midguts of GFP and pJNK staining in control flies and 7% DSS stimulated flies supplemented with or without 1 µM SIL. pJNK: red, a marker that indicates the activated JNK signaling pathway. Scale bars represent 10 µm. **(D)** Quantitation of pJNK fluorescence intensity per cell (*n* = 184, 196, 171). **(E)** Quantification of relative mRNA expression of the JNK signal-regulated genes (*Mmp1*, *MtnA*, and *dpp*) in the midguts of control flies and 7% DSS stimulated flies supplemented with or without 1 µM SIL. **(F–H)** Immunofluorescence images of *Drosophila* posterior midguts of GFP and Dl staining in 7% DSS stimulated flies. *esg* ^*ts*^ *-Gal4*-driven: *UAS-lacZ*, *USA-bsk* ^*DN*^ without SIL, and *USA-bsk* ^*DN*^ with 1 µM SIL. Scale bars represent 25 µm. **(I,J)** Quantification of the ratio of *esg*-GFP positive cells and Dl positive cells to DAPI positive cells per ROI (*n* = 30, 38, 42). **(K)** Quantification of the number of pH3 positive cells in the whole guts of flies (*n* = 19, 22, 24). Data are represented as means ± SD. Student’s *t*-tests, ns represents *p* > 0.05, ****p < 0.0001.

To investigate whether SIL alleviates intestinal inflammation caused by DSS by regulating the JNK signaling pathway, we detected the activity of JNK signaling by staining the phosphorylated JNK (pJNK). We found that pJNK expression was significantly increased when the *Drosophila* intestine was subjected to DSS-induced injury (Figures 5A, B, D), and supplementation with SIL inhibited the activation of the JNK signaling pathway (Figures 5C, D). Besides, RT-qPCR analyses revealed that SIL administration decreased the expression of JNK signal-regulated genes (*Mmp1*, *MtnA*, and *dpp*) in DSS-stimulated *Drosophila* midgut (Figure 5E).

To further validate the effect of SIL on the JNK signaling pathway, we inhibited JNK signaling in ISCs by overexpressing the dominant-negative form of *basket* (*bsk* ^*DN*^, *bsk* is a key component of the JNK signaling pathway (Tafesh-Edwards and Eleftherianos, 2020). In *esg* ^*ts*^-driven *bsk* ^*DN*^ flies, we found that the number of *esg*-GFP^+^, Dl^+^, and pH3^+^ cells were all reduced when subjected to DSS injury compared to the control group (Figures 5F, G, I–K). In addition, SIL supplementation failed to further reduce the number of *esg*-GFP^+^, Dl^+^, and pH3^+^ cells based on inhibition of JNK signaling (Figures 5H–K), which suggested that SIL alleviates *Drosophila* intestinal inflammation by inhibiting the JNK signaling pathway.

### 3.6 SIL inhibits DSS-induced ISC overproliferation by suppressing the EGFR signaling pathway

**FIGURE 6:**
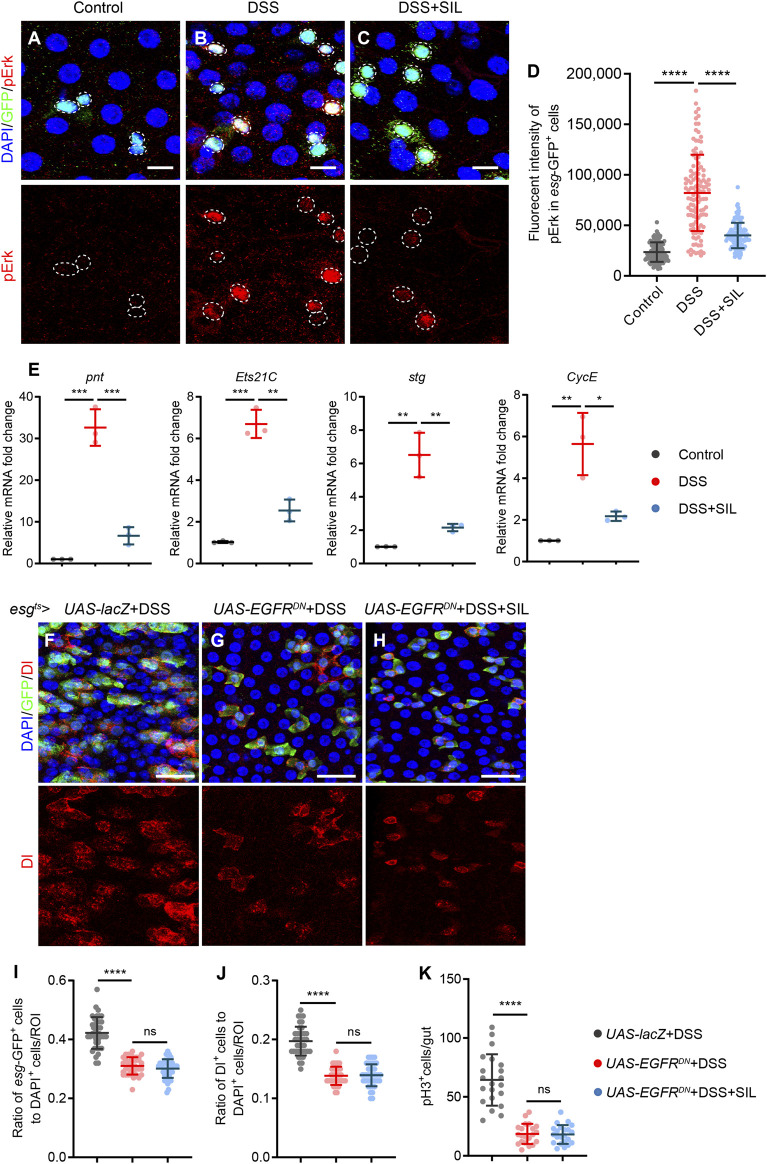
SIL inhibits the EGFR signaling pathway. **(A–C)** Immunofluorescence images of *Drosophila* (*esg*-GFP/CyO) posterior midguts of GFP and pErk staining in control flies and 7% DSS stimulated flies supplemented with or without 1 µM SIL. pErk: red, a marker indicates the activated EGFR signaling pathway. Scale bars represent 10 µm. White circles indicate GFP positive cells and pErk staining. **(D)** Quantitation of pErk fluorescence intensity in *esg*-GFP positive cells (n = 110, 116, 110). **(E)** Quantification of relative mRNA expression of the EGFR signal-regulated genes (*pnt*, *Ets21C*, *stg*, and *CycE*) in the midguts of control flies and 7% DSS stimulated flies supplemented with or without 1 µM SIL. **(F–H)** Immunofluorescence images of *Drosophila* posterior midguts of GFP and Dl staining in 7% DSS stimulated flies. *esg* ^*ts*^ *-Gal4*-driven: *UAS-lacZ*, *USA-EGFR* ^*DN*^ without SIL, and *USA-EGFR* ^*DN*^ with 1 µM SIL. Scale bars represent 25 µm. **(I,J)** Quantification of the ratio of *esg*-GFP positive cells and Dl positive cells to DAPI positive cells per ROI (n = 39, 37, 41). **(K)** Quantification of the number of pH3 positive cells in the whole guts of flies (n = 21, 21, 22). Data are represented as means ± SD. Student’s *t*-tests, ns represents *p* > 0.05, ****p < 0.0001.

EGFR signaling has been widely demonstrated to be involved in regulating the proliferation of ISCs and is downstream of JNK signaling (Jiang et al., 2011; Jiang et al., 2016). Therefore, we investigated whether SIL inhibits the DSS-induced overproliferation of ISCs by regulating the EGFR signaling pathway. Firstly, we determined the activation of EGFR signaling by detecting the expression of pErk (an indicator of EGFR signaling activation) in *esg*-GFP^+^ cells. We found that the expression of pErk was significantly elevated in *esg*-GFP^+^ cells when the *Drosophila* intestine was damaged by DSS (Figures 6A, B, D), and SIL supplementation can suppress the activation of EGFR signaling (Figures 6C, D). We performed RT-qPCR analyses and found that SIL administration decreased the expression of EGFR signal-regulated genes (*pnt*, *Ets21C*, *stg* and *CycE*) in DSS-stimulated *Drosophila* midgut (Figure 6E).

Next, we overexpressed the dominant-negative form of *EGFR* (*EGFR* ^*DN*^) to further verify whether SIL inhibits EGFR signaling. If SIL inhibits the DSS-induced ISC overproliferation by repressing the EGFR signaling pathway, then supplementing SIL in the *esg* ^*ts*^-driven *EGFR* ^*DN*^ *Drosophila* would have no additional effect. As expected, the number of *esg*-GFP^+^, Dl^+^, and pH3^+^ cells in the *EGFR* ^*DN*^ *Drosophila* increased insignificantly when the intestine was damaged by inflammation (Figures 6F, G, I–K), and SIL supplementation did not further reduce the number of *esg*-GFP^+^, Dl^+^, and pH3^+^ cells (Figures 6H–K). These results demonstrated that SIL inhibits DSS-induced ISC overproliferation by suppressing the EGFR signaling pathway.

## 4 Discussion

**FIGURE 7:**
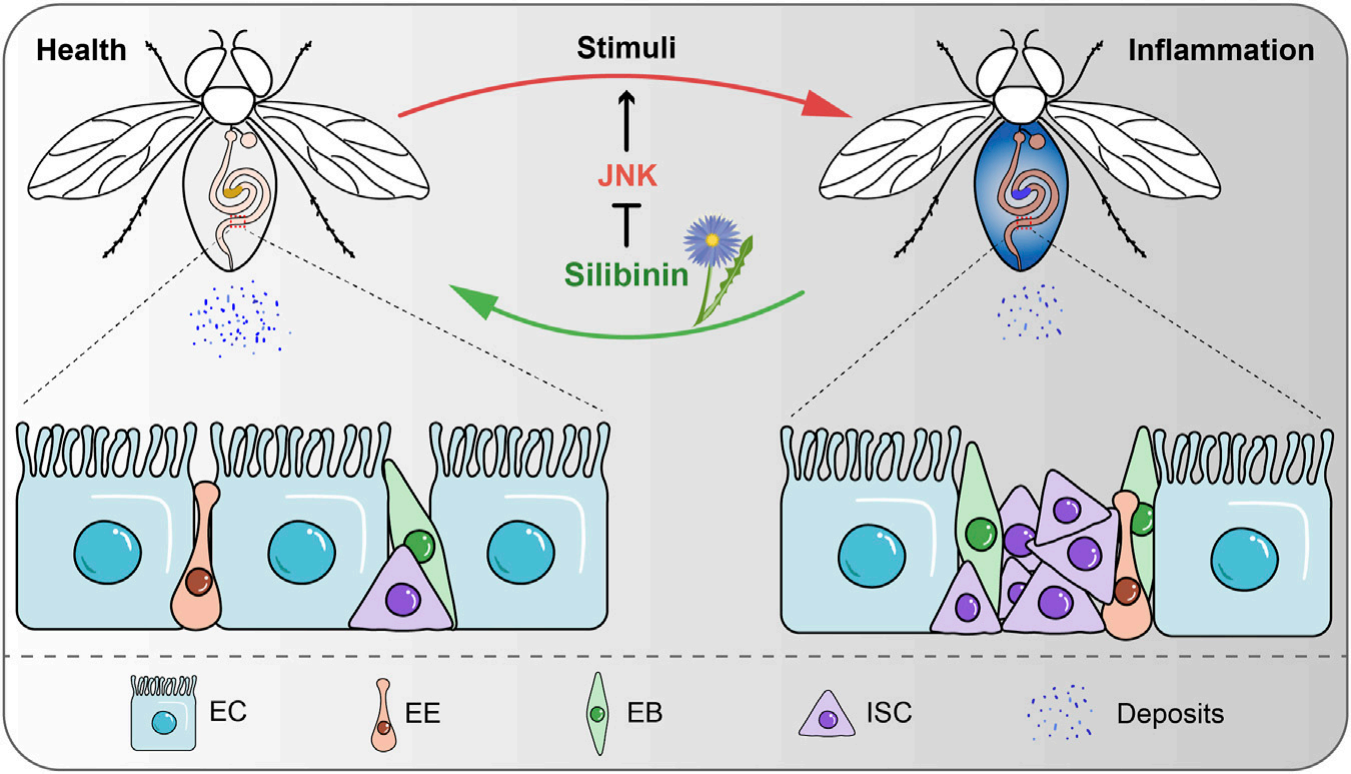
Schematic model for SIL alleviates intestinal inflammation in *Drosophila*. SIL inhibits the overproliferation of ISCs, improves intestinal function and attenuates intestinal inflammatory injury by inhibiting the JNK signaling pathway.

Inflammation is the basis of a variety of physiological and pathological processes, mainly induced by infection and tissue damage (Medzhitov, 2008). However, excessive and chronic inflammation can trigger harmful immune processes that lead to multiple diseases, including cancer, atherosclerosis, neurodegenerative diseases, and IBDs (Iyengar et al., 2016; Ransohoff, 2016; Wolf and Ley, 2019; Rimola et al., 2022). The intestine is the main organ for nutrient digestion and absorption and the primary site of the immune response (Mowat and Agace, 2014). IBDs are characterized by recurrent inflammation and mucosal damage, which severely disrupts the integrity of intestinal epithelium and intestinal functions (Ramos and Papadakis, 2019). For many years, researchers have been working to find effective treatment strategies to relieve symptoms and improve the quality of life of patients with IBDs. In this study, we have confirmed that silibinin alleviates intestinal inflammation in *Drosophila* by inhibiting the JNK signaling pathways (Figure 7).

*Drosophila melanogaster* is an excellent model organism that has been widely used in mechanism research and drug screening of various diseases (Galikova and Klepsatel, 2018; Souidi and Jagla, 2021; Yan et al., 2022). The similarity of its intestinal function and the high conservation of signaling pathways to mammals make *Drosophila* an attractive model for studying intestinal inflammatory diseases (Medina et al., 2022; Wei et al., 2022). Herein, our results demonstrated that SIL supplementation can protect ISCs from hyperproliferation caused by DSS or BLM. Arm staining and smurf assay indicated that SIL protects the intestinal epithelial barrier of *Drosophila* when stimulated by DSS-induced inflammation. In addition, SIL improves the intestinal functions of *Drosophila*, including acid-base homeostasis in the CCR and intestinal excretion function. ROS accumulation is closely associated with the development of intestinal inflammatory diseases (Aviello and Knaus, 2017). We found that SIL supplementation reduces ROS levels and decreases the expression of antioxidant-related genes and inflammatory cytokines. Antimicrobial peptides (AMPs) are important immunoreactive proteins with antibacterial and antiviral activities, and their production is an important defense mechanism in *Drosophila* (Hanson and Lemaitre, 2020; Lazzaro et al., 2020). In this study, we found that SIL supplementation decreases the expression of AMPs in *Drosophila* with DSS stimulation. Furthermore, our results suggested that SIL could prolong the lifespan of *Drosophila* under DSS or BLM stimulation. However, since these stimuli can cause damage to other tissues and SIL can also act outside the intestine, we cannot exclude that the lifespan-prolonging effect of SIL is generated through other mechanisms, which remains to be verified by further studies.

The JNK signaling pathway is an evolutionarily conserved kinase cascade composed of distinct mitogen-activated protein kinases (MAPKs) (Zeke et al., 2016), which is activated in response to a variety of stimuli, including DNA damage, oxidative stress, infection, and inflammatory cytokines (Tournier et al., 2001; Yoshida et al., 2005; Tafesh-Edwards and Eleftherianos, 2020). While mammals express three JNK proteins (JNK1/2/3, also known as MAPK8/9/10), *Drosophila* expresses only one JNK protein encoded by *basket* (*bsk*) (Chimnaronk et al., 2015). In *Drosophila*, JNK signaling plays a key role in various biological processes such as cell proliferation, differentiation, apoptosis, stress responses, and immunity (Tare et al., 2016; Herrera and Bach, 2021; Mishra et al., 2021; Yu et al., 2022). In this study, through network pharmacological analysis and molecular docking, we found that the target of SIL in intestinal inflammatory diseases is the JNK signaling pathway. Through the activity detection of JNK signaling and the overexpression of the dominant-negative form of *bsk* in ISCs, we further confirmed that SIL alleviates DSS-induced intestinal inflammation by inhibiting the JNK signaling pathway in *Drosophila*.

The EGFR pathway is a mitogenic signaling pathway that is required for the activation of ISC division during *Drosophila* intestinal regeneration (Buchon et al., 2010; Jiang et al., 2011). However, aberrant activation of EGFR signaling induces a dramatic proliferation of ISCs, leading to intestinal hyperplasia in *Drosophila* (Jiang et al., 2011). Studies have shown that EGFR signaling can be regulated by multiple signaling pathways, including the Hippo, Hedgehog, and JNK pathways (Ren et al., 2010; Jiang et al., 2016). Our results demonstrated that SIL supplementation suppresses DSS-induced ISC overproliferation by inhibiting the aberrant activation of EGFR signaling. However, since the development and progression of IBDs are closely associated with dysbiosis of the intestinal microbiota and SIL has been reported to possess a good antibacterial activity (Lee et al., 2003; Ni et al., 2017; Vimalraj et al., 2018; Lee and Chang, 2021), and our results also showed that supplementation with SIL reduced the expression of AMPs in the *Drosophila* intestine. We cannot disregard the possibility that the alleviative effect of SIL on intestinal inflammation in *Drosophila* is achieved through modulation of the gut microbiota composition, further investigation is required to elucidate this aspect.

In summary, when the *Drosophila* intestine experienced damage from stressful stimuli, supplementation of SIL showed the ability to inhibit the excessive proliferation of ISCs, enhance intestinal function, mitigate inflammatory injury to the intestine, and prolong lifespan. Additionally, this study provides evidence that SIL alleviates DSS-induced intestinal inflammation by inhibiting the JNK signaling pathway.

## Data Availability

The original contributions presented in the study are included in the article/Supplementary Material, further inquiries can be directed to the corresponding author.
