# Supplementary material for: Silibinin alleviates intestinal inflammation via inhibiting JNK signaling in Drosophila
Source: Front Pharmacol. 2023 Sep 14;14:1246960. doi: 10.3389/fphar.2023.1246960 (PMC10539474; doi:10.3389/fphar.2023.1246960)
Supplement: Supplementary file 1 [file DataSheet1.docx]

Supplementary Material

Silibinin Alleviates Intestinal Inflammation via Inhibiting JNK Signaling in *Drosophila*

La Yan^1, 2†^, Juanyu Zhou^1†^, Lu Yuan^3†^, Jinbao Ye^1^, Xudong Zhao^2^, Gang Ren^3^, and Haiyang Chen^1*^

*** Correspondence:** Haiyang Chen: [chenhy82@scu.edu.cn](mailto:chenhy82@scu.edu.cn)

# Supplementary Figures and Tables

## Supplementary Figures


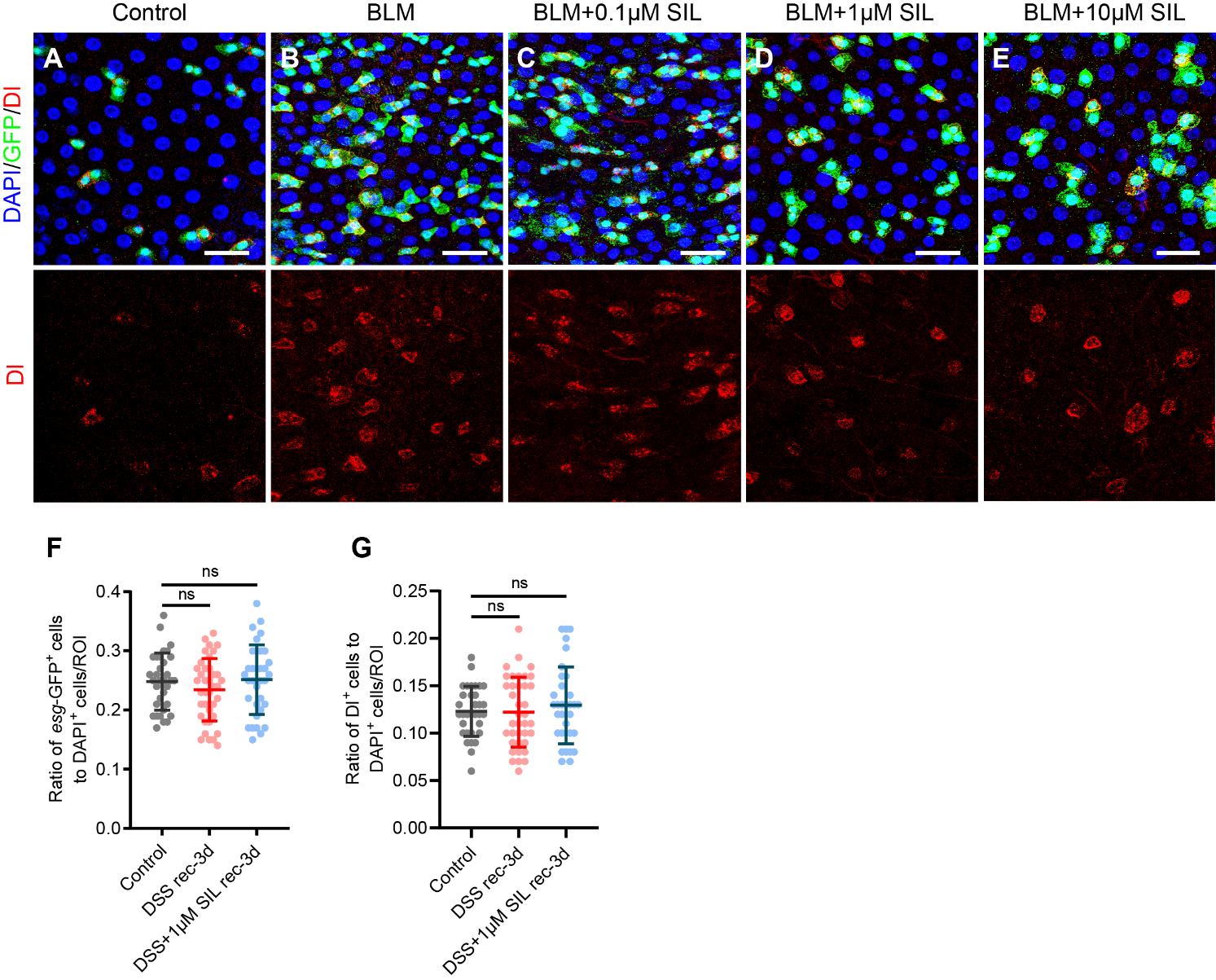


**Supplementary Figure S1.** SIL did not affect the final repair of the intestine.  **(A-E)** Immunofluorescence images of *Drosophila* (*esg-GFP/CyO*) posterior midguts of GFP and Dl staining in control flies and 25 µg/mL BLM stimulated flies supplemented with or without SIL. Three concentrations of SIL were studied: 0.1, 1, and 10 µM. GFP: green, ISCs and progenitor cells marker; Dl: red, ISCs marker; DAPI: blue, nuclei. Scale bars represent 25 µm. **(F)** Quantification of the ratio of *esg*-GFP positive cells to DAPI positive cells per ROI (n = 33, 38, 34 from left to right). **(G)** Quantification of the ratio of Dl positive cells to DAPI positive cells per ROI (n = 33, 38, 34 from left to right).


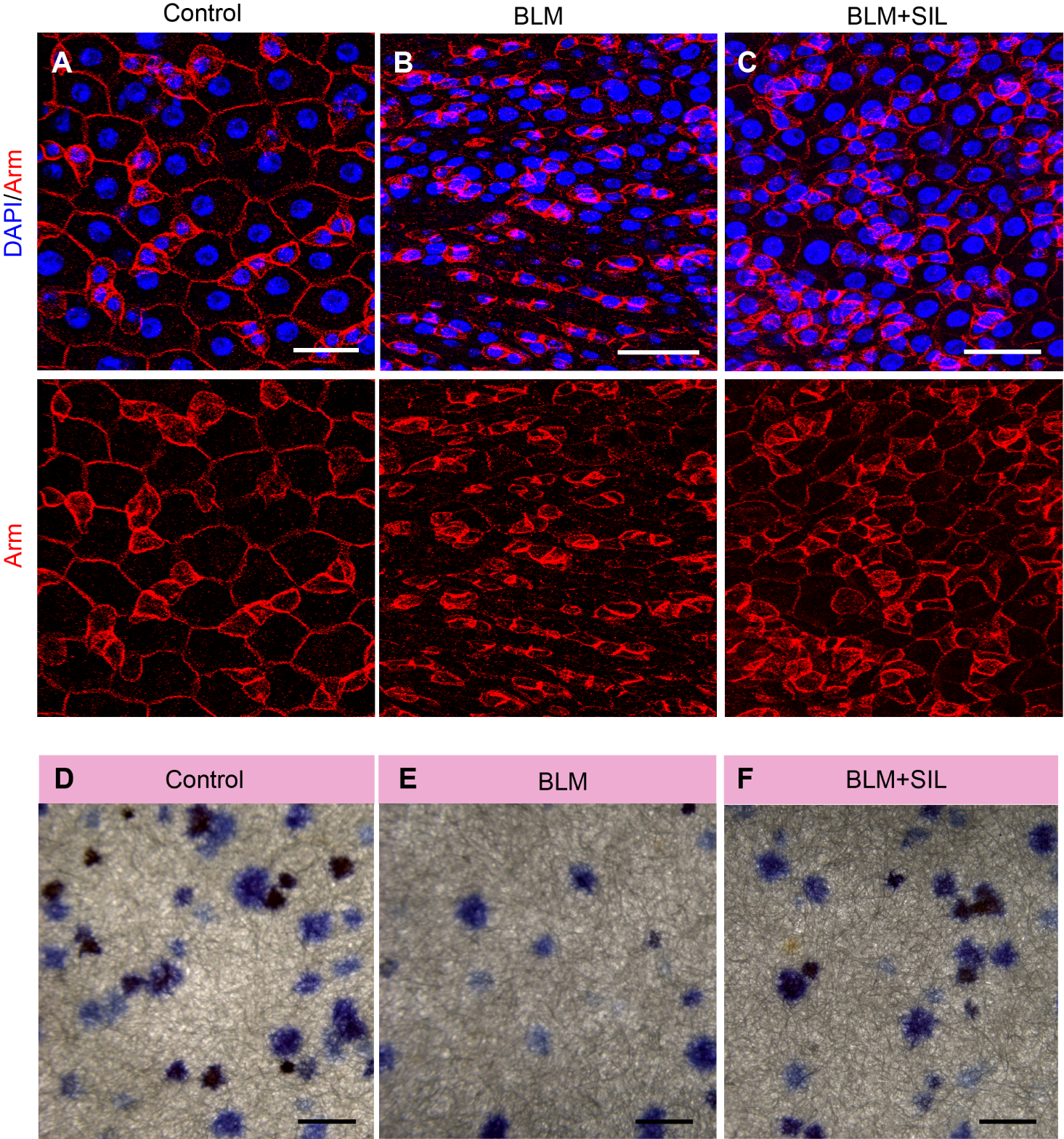


**Supplementary Figure S2.** SIL prevents BLM-induced intestinal dysfunction. **(A-C)** Immunofluorescence images of *Drosophila* (*esg-GFP/CyO*) posterior midguts of Armadillo (Arm) staining in control flies and 25 µg/mL BLM stimulated flies supplemented with or without SIL. Arm: red, cell membrane maker. Scale bars represent 25 µm. **(D-E)** Representative images of excretion deposits in control flies and 25 µg/mL BLM stimulated flies supplemented with or without SIL. Scale bars represent 2 mm.

## Supplementary Tables

| **Supplementary Table S1.** The list of the antibodies | | |
| --- | --- | --- |
| Reagent | Source | Dilution |
| Chicken polyclonal anti-GFP | Abcam Cat# ab13970 RRID:AB_300798 | 1: 1000 |
| Mouse anti-Delta | DSHB Cat# C594.9B RRID:AB_528194 | 1: 50 |
| Rabbit anti-phosphoHistone H3 (Ser10) | Millipore Cat# 06-570 RRID:AB_310177 | 1: 1000 |
| Mouse anti-Armadillo | DSHB Cat# N2 7A1 Armadillo  RRID:AB_528089 | 1: 50 |
| Mouse anti-pJNK | Cell Signaling Cat # 9255 | 1: 150 |
| Rabbit anti-pERK | Cell Signaling Cat #4370 | 1: 100 |

| **Supplementary Table S2.** The list of the primer sequences | |
| --- | --- |
| Target | Sequence |
| *Cat* | F: 5’-TTCCTGTGGGCAAAATGGTG-3’  R: 5’-ATCTTCACCTTGTACGGGCA-3’ |
| *SOD* | F: 5’-CAAGGGCACGGTTTTCTTC-3’  R: 5’-CCTCACCGGAGACCTTCAC-3’ |
| *GstD1* | F: 5’-CATCGCGAGTTTCACAACAG-3’  R: 5’-GTTGAGCAGCTTCTTGTTCAG-3’ |
| *Attacin A* | F: 5’-GCATCCTAATCGTGGCCCT-3’  R: 5’-AGCGGGATTGGAGGTTAAGG-3’ |
| *Cecropin C* | F: 5’-GCATTGGACAATCGGAAGCC-3’  R: 5’-GCGCGTTATCCTGGTAGAGT-3’ |
| *Defensin* | F: 5’-CTCGTGGCTATCGCTTTTGC-3’  R: 5’-CCACTTGGAGAGTAGGTCGC-3’ |
| *Diptericin* | F: 5’-CTCAATCTTCAGGGAGGCGG-3’  R: 5’-AGGTGCTTCCCACTTTCCAG-3’ |
| *upd2* | F: 5’- CGGAACATCACGATGAGCGAAT-3’  R: 5’- TCGGCAGGAACTTGTACTCG-3’ |
| *upd3* | F: 5’- ATCCCACCAATCCCCTGAAG-3’  R: 5’- AGATTGCAGGTGTTCTCCCA-3’ |
| *Mmp1* | F: 5’- AGGACTCCAAGGTAGACACAC-3’  R: 5’- TTGCCGTTCTTGTAGGTGAACGC-3’ |
| *MtnA* | F: 5’- TGCAAATGCGCCAGCCAG-3’  R: 5’- TCGGAGCAGCCGCAGG-3’ |
| *dpp* | F: 5’- CGAACCGACAGTTCAACGAAG-3’  R: 5’- GCTCCTCTGCTCCTTGACAG-3’ |
| *pnt* | F: 5’- ACGCCCTATGATGCTCAATC-3’  R: 5’- TATCCAGACCCAAGGTGCTC-3’ |
| *Ets21C* | F: 5’- CCGGGCACTCAGGTACTACT-3’  R: 5’- CATACTGGAGGCCGGATCT-3’ |
| *stg* | F: 5’- GAGCTGATGGGTCTGCTCTC-3’  R: 5’- ATGTGGAGGACAGGCTGTTG-3’ |
| *CycE* | F: 5’- ACAAATTTGGCCTGGGACTA-3’  R: 5’- GGCCATAAGCACTTCGTC-3’ |
| *Rp49* | F: 5’-ATCGGTTACGGATCGAACAAGC-3’ R: 5’-GTAAACGCGGTTCTGCATGAGC-3’ |
